# A Rare Case of D-transposition of the Great Arteries (TGA) With Ventricular Septal Defect (VSD), Dysplastic Pulmonary Valve (Absent Pulmonary Valve Physiology), and Aortic Valve Stenosis in a Term Neonate

**DOI:** 10.7759/cureus.100389

**Published:** 2025-12-30

**Authors:** Ling Ai Soon, Mohammad Tamim

**Affiliations:** 1 Pediatric Cardiology Department, Hospital Pulau Pinang, George Town, MYS

**Keywords:** absent pulmonary valve, aortic stenosis, congenital heart disease, dysplastic pulmonary valve, neonatal cyanosis, transposition of the great arteries, ventricular septal defect

## Abstract

Absent pulmonary valve (APV) syndrome is an uncommon congenital cardiac anomaly most frequently associated with tetralogy of Fallot, representing only 3% of such cases. Its occurrence with transposition of the great arteries (TGA) is exceptionally rare, and the presence of coexisting aortic valve stenosis adds significant hemodynamic complexity. We describe a term neonate who presented immediately after birth with severe cyanosis and desaturation, requiring intubation and prostaglandin E₁ infusion. Chest radiography demonstrated marked cardiomegaly, while echocardiography confirmed TGA with a large ventricular septal defect (VSD), a dysplastic pulmonary valve resulting in free pulmonary regurgitation with massive main pulmonary artery dilatation, hypoplastic branch pulmonary arteries, and moderate aortic valve stenosis. Given the complexity of the cardiac anatomy, the guarded prognosis, and significant limitations in social support, including the poor socioeconomic status of very young parents who lived far from the nearest tertiary hospital, the multidisciplinary team elected to pursue conservative management. This rare constellation of cardiac lesions underscores the challenges of early recognition, risk stratification, and decision-making in neonates with severe conotruncal malformations.

## Introduction

Transposition of the great arteries (TGA) is a major conotruncal anomaly characterized by ventriculoarterial discordance. The coexistence of a ventricular septal defect (VSD) is relatively common; about one-third to 40% of patients with d-TGA have an associated ventricular septal defect occurring in 30%-40% of cases [1]. In contrast, absent pulmonary valve (APV) syndrome is a rare congenital cardiac malformation characterized by absent, dysplastic, or rudimentary pulmonary valve leaflets. It is classically associated with tetralogy of Fallot (TOF) and is only rarely reported in conjunction with TGA. When present, the dysplastic or rudimentary pulmonary valve results in severe pulmonary regurgitation and aneurysmal dilatation of the main pulmonary artery (MPA) and its branches. The combination of TGA, VSD, and APV is exceedingly rare, with only a few cases reported in the literature, including those described by Sakai et al. [2]. Furthermore, the presence of concomitant aortic stenosis in this constellation is exceptionally uncommon, with no previously reported cases to date. This case highlights an extraordinarily rare and complex cardiac anatomy, adding to the limited existing data and underscoring the diagnostic challenges and management dilemmas encountered in neonatal practice.

## Case presentation

We report the case of a term, non-syndromic newborn whose mother had an uncomplicated pregnancy. Immediately following birth, the infant was noted to be cyanotic with poor respiratory effort and an oxygen saturation of 80% despite supplemental oxygen administration. On physical examination, the neonate appeared markedly cyanosed, and a loud pansystolic murmur was audible along the left sternal border. The remainder of the systemic examination was unremarkable.

**Figure 1:**
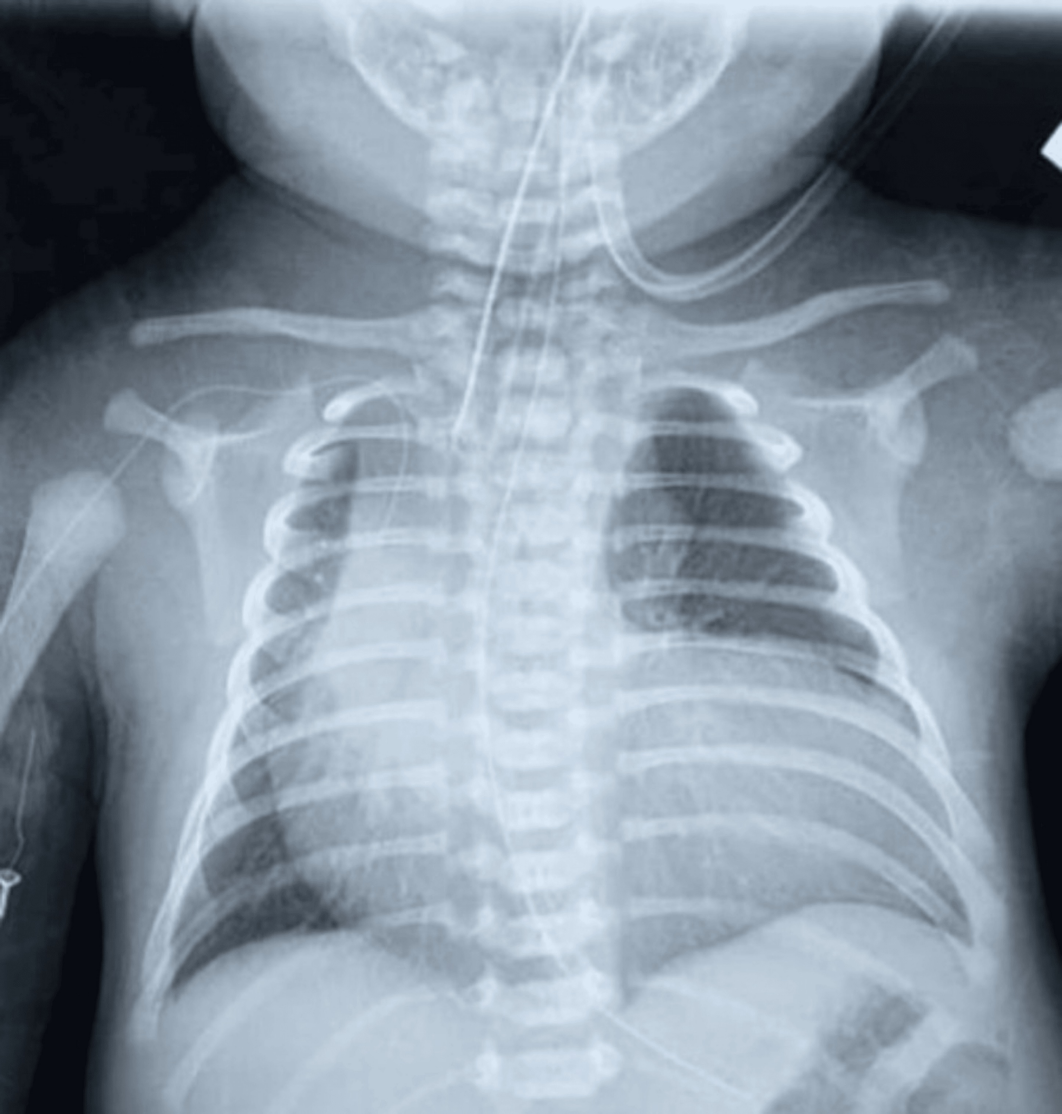
Radiographic evaluation showed marked cardiomegaly and a boot-shaped cardiac configuration. Furthermore, there was evidence of increased pulmonary vascular markings (pulmonary plethora), suggesting pulmonary overcirculation.

The neonate required immediate intubation and was commenced on a prostaglandin E₁ infusion (10 ng/kg/min) to maintain ductal patency following an urgent bedside echocardiogram. Mechanical ventilation was initiated using low ventilatory settings, and the patient did not require inotropic support. A chest radiograph demonstrated gross cardiomegaly (Figure 1). Following discussion with the pediatric cardiology team, a CT pulmonary angiogram (CTPA) was planned to further delineate the anatomy and facilitate multidisciplinary discussions with the cardiothoracic surgery team regarding surgical feasibility.

Echocardiography

Figures 2-10 illustrate the echocardiographic and CT findings of a term neonate with D-transposition of the great arteries, large ventricular septal defect, dysplastic pulmonary valve, and aortic stenosis.

**Figure 2:**
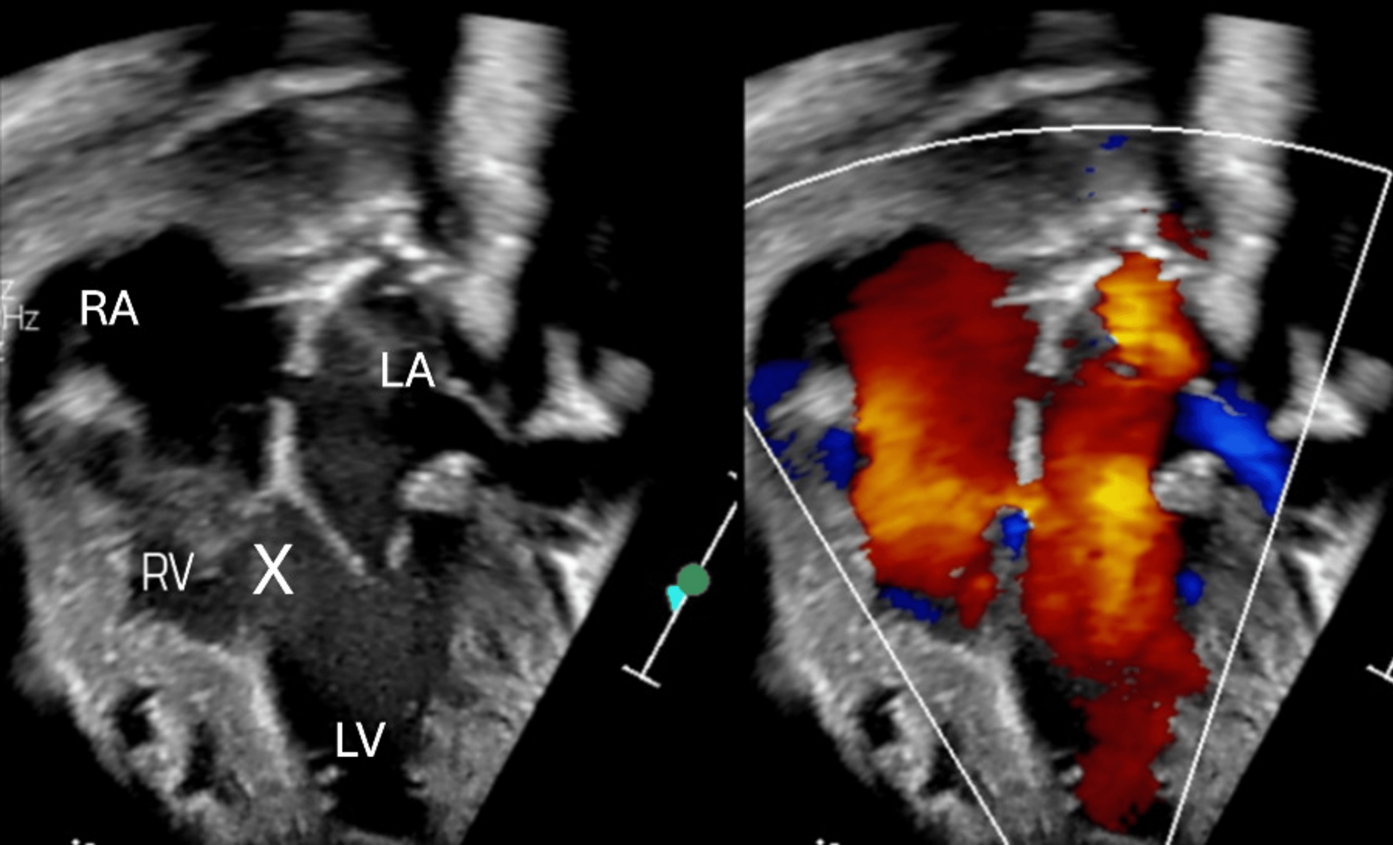
Four-chamber view demonstrating a hypertrophied right ventricle (RV) and a large ventricular septal defect (label as X). RA: right atrium, LA: left atrium, LV: left ventricle.

**Figure 3:**
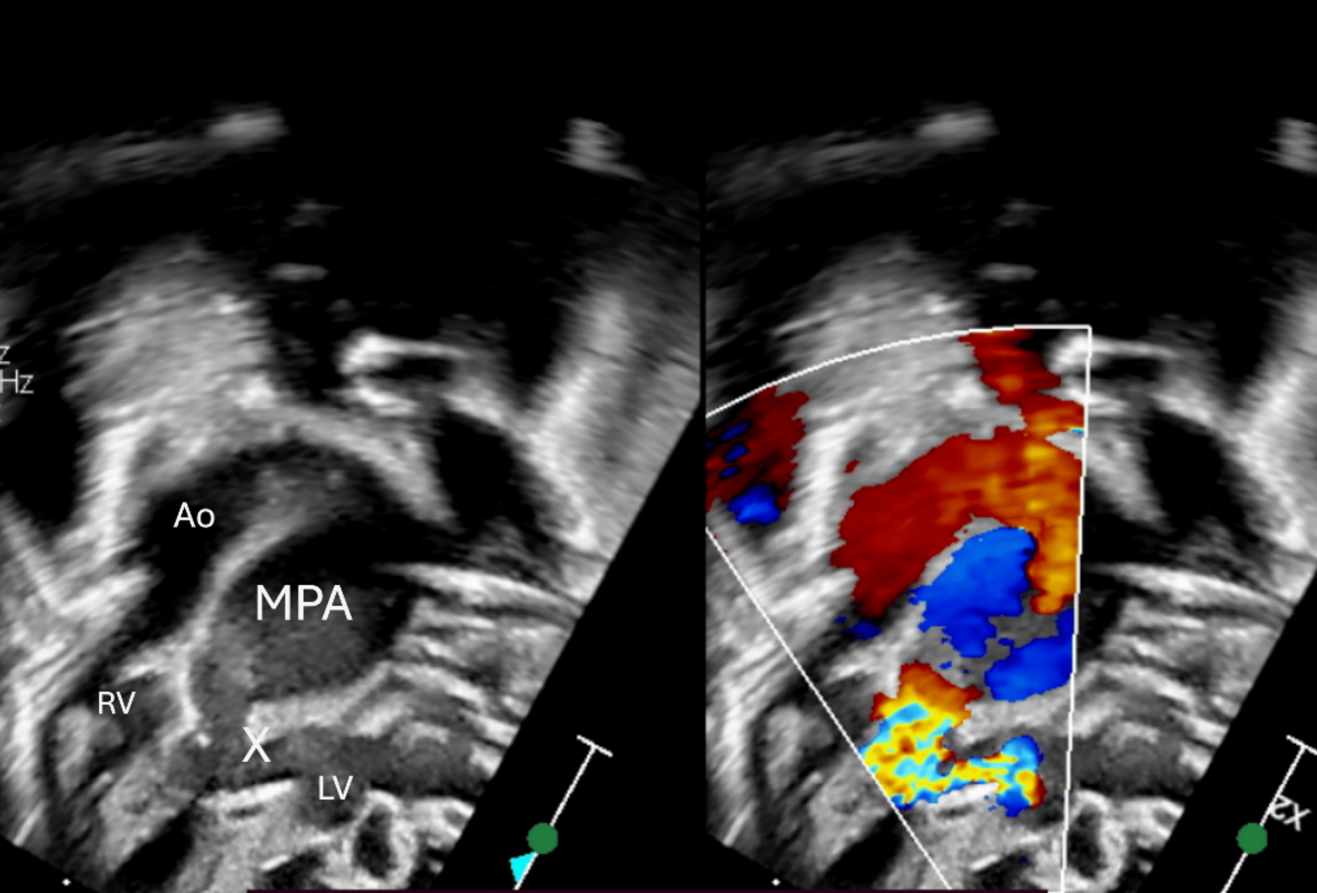
Dysplastic and rudimentary pulmonary valve (label as X) with massively dilated main pulmonary artery (MPA) and free flow pulmonary regurgitation. Ao: aorta, RV: right ventricle, LV: left ventricle.

**Figure 4:**
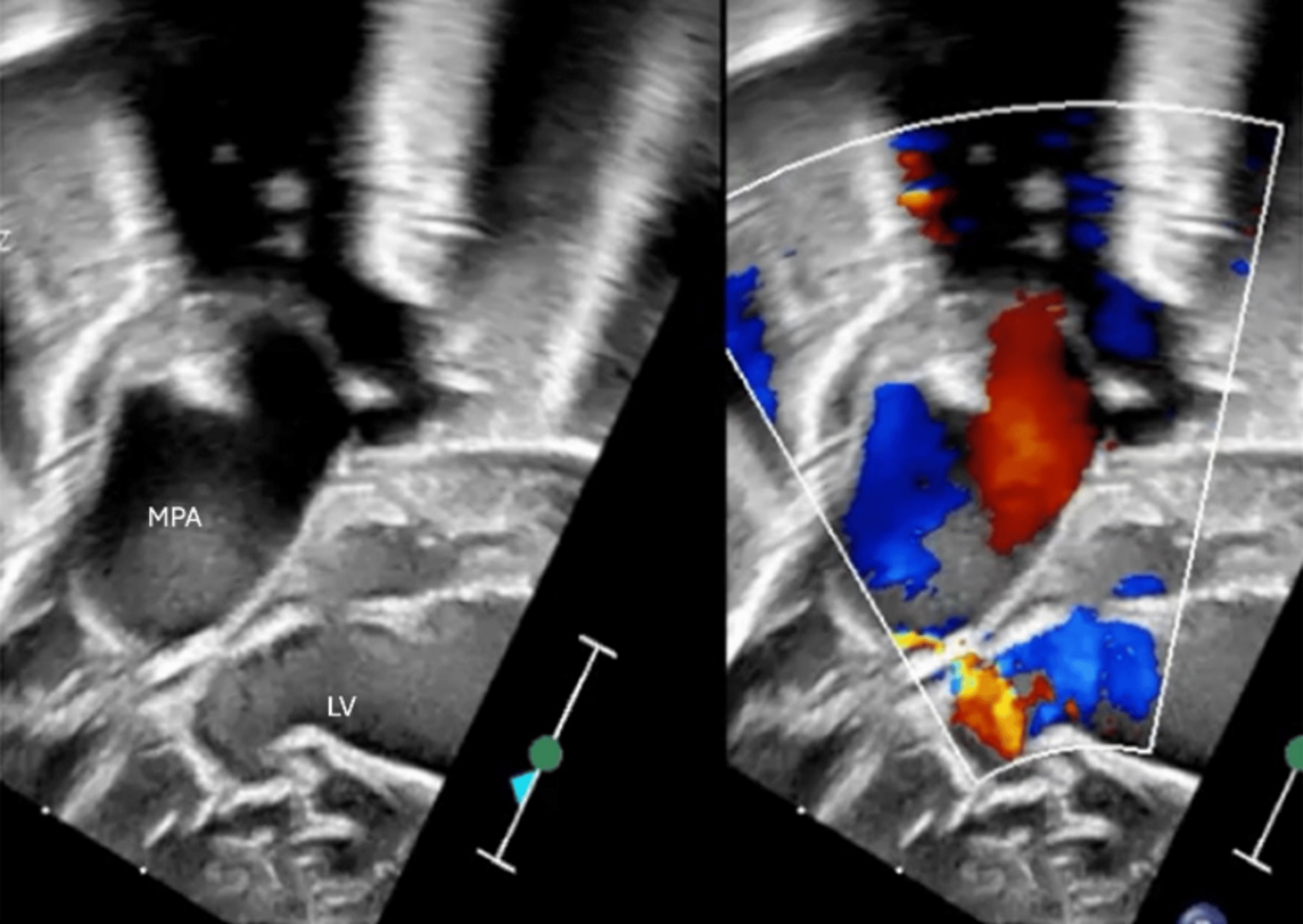
Hugely dilated main pulmonary artery (MPA) and its branches. LV: left ventricle.

**Figure 5:**
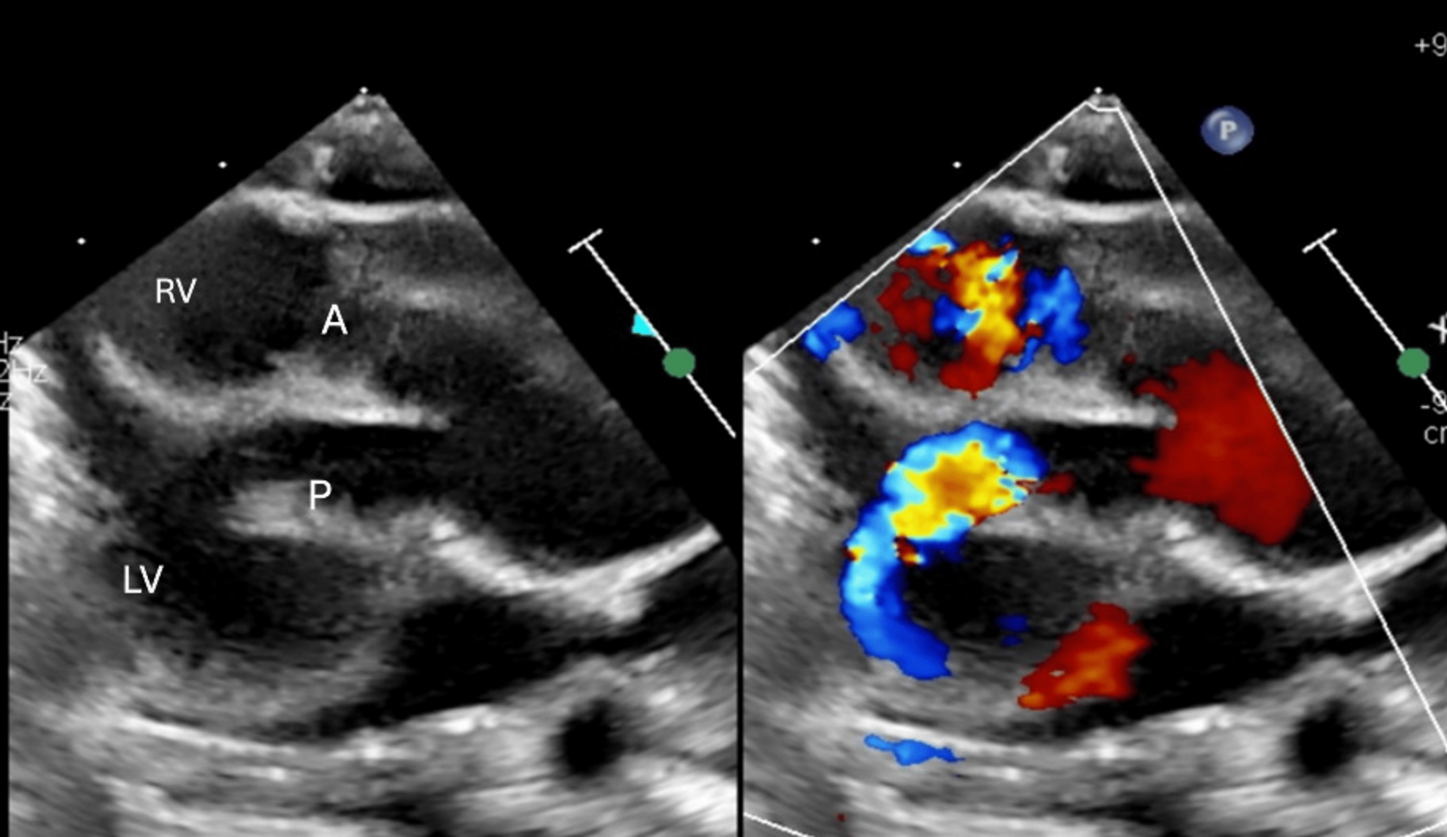
Parallel orientation of the great arteries, with the aorta (A) positioned anterior to the pulmonary (P) artery. LV: left ventricle, RV: right ventricle.

**Figure 6:**
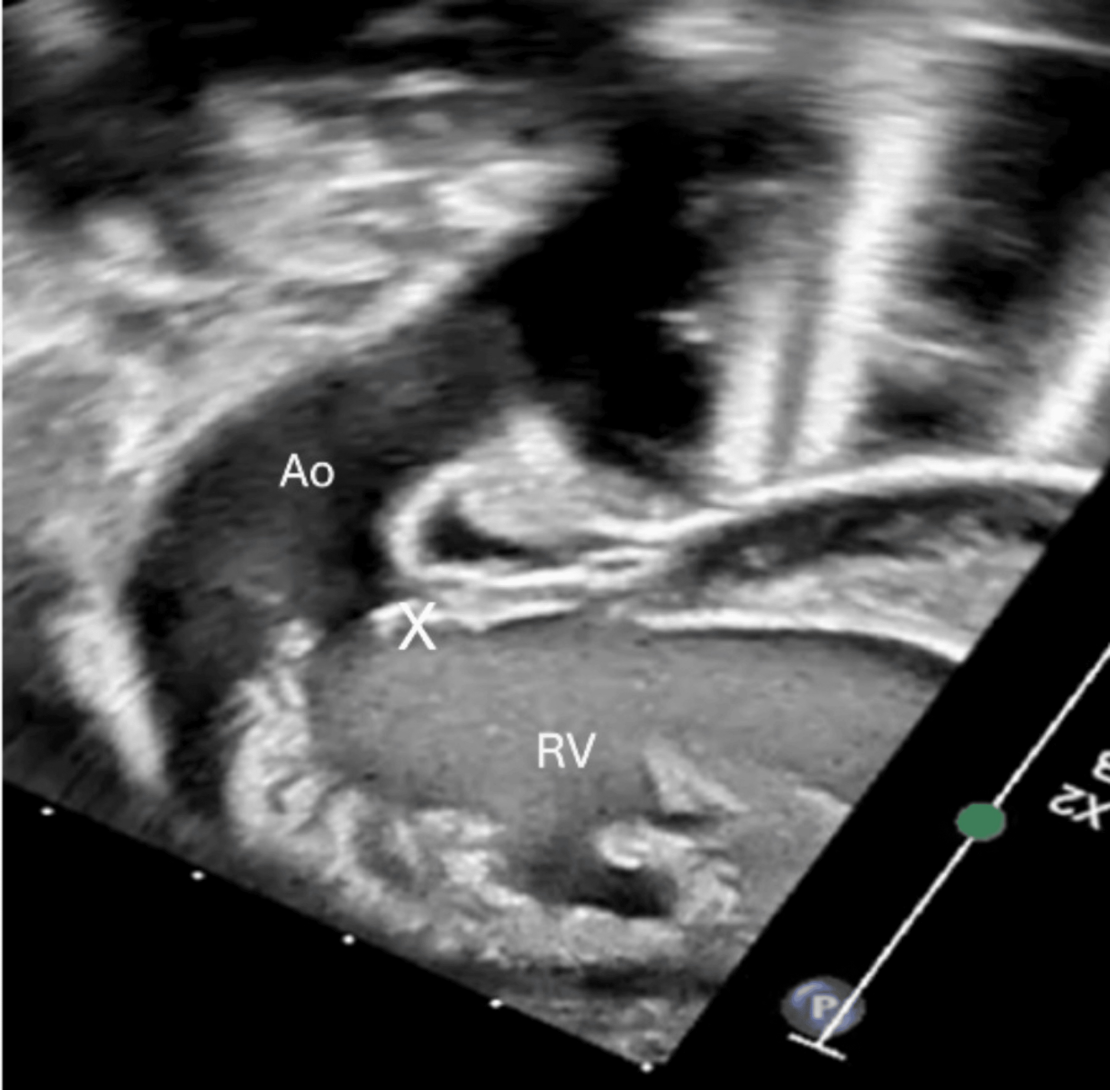
Aorta arising from right ventricle (RV) with thickened aortic valve (X). Ao: aorta.

**Figure 7:**
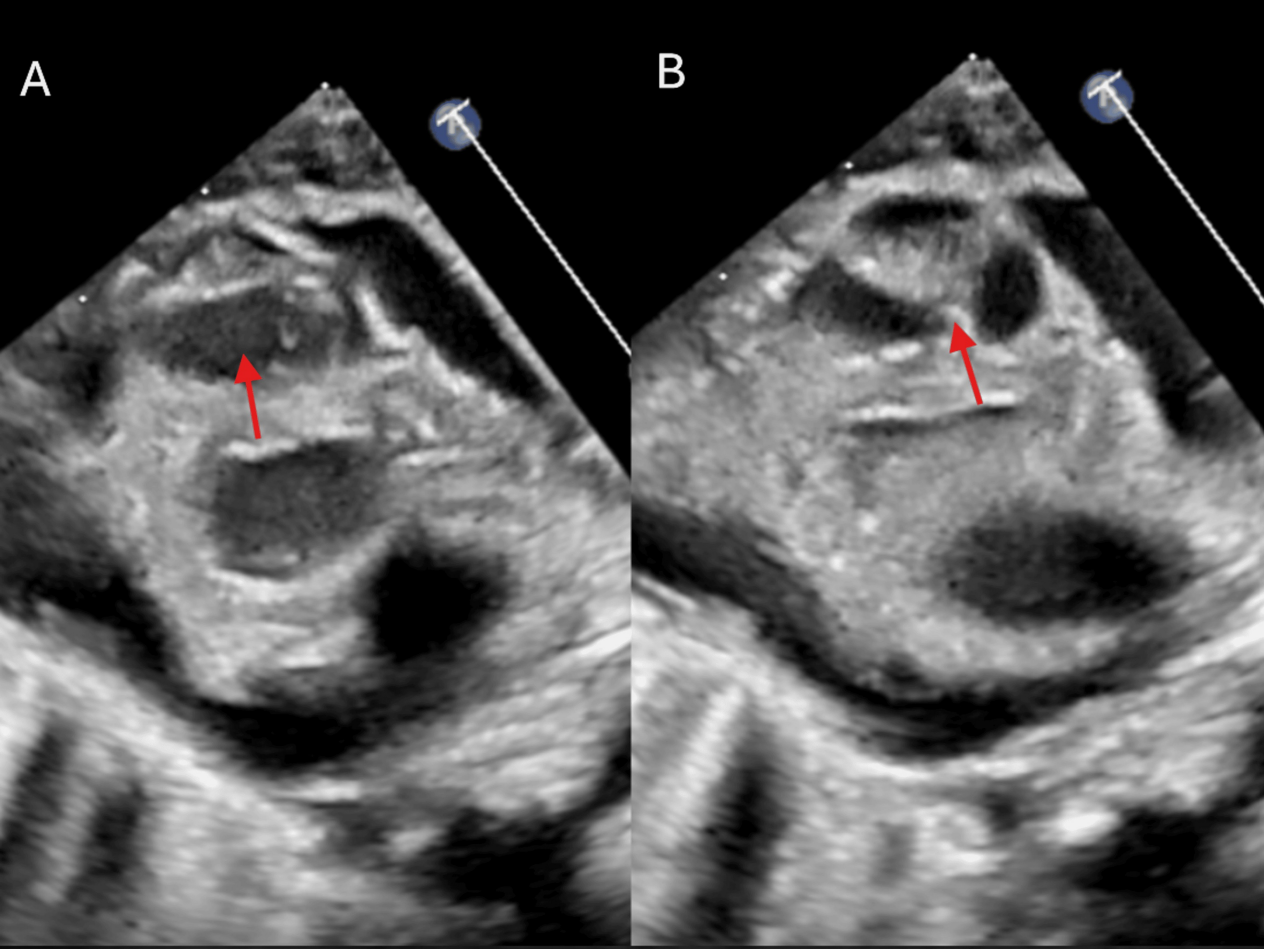
Parasternal long-axis view demonstrating thickening of the aortic valve leaflets, evident during both (A) systole and (B) diastole (arrow).

**Figure 8:**
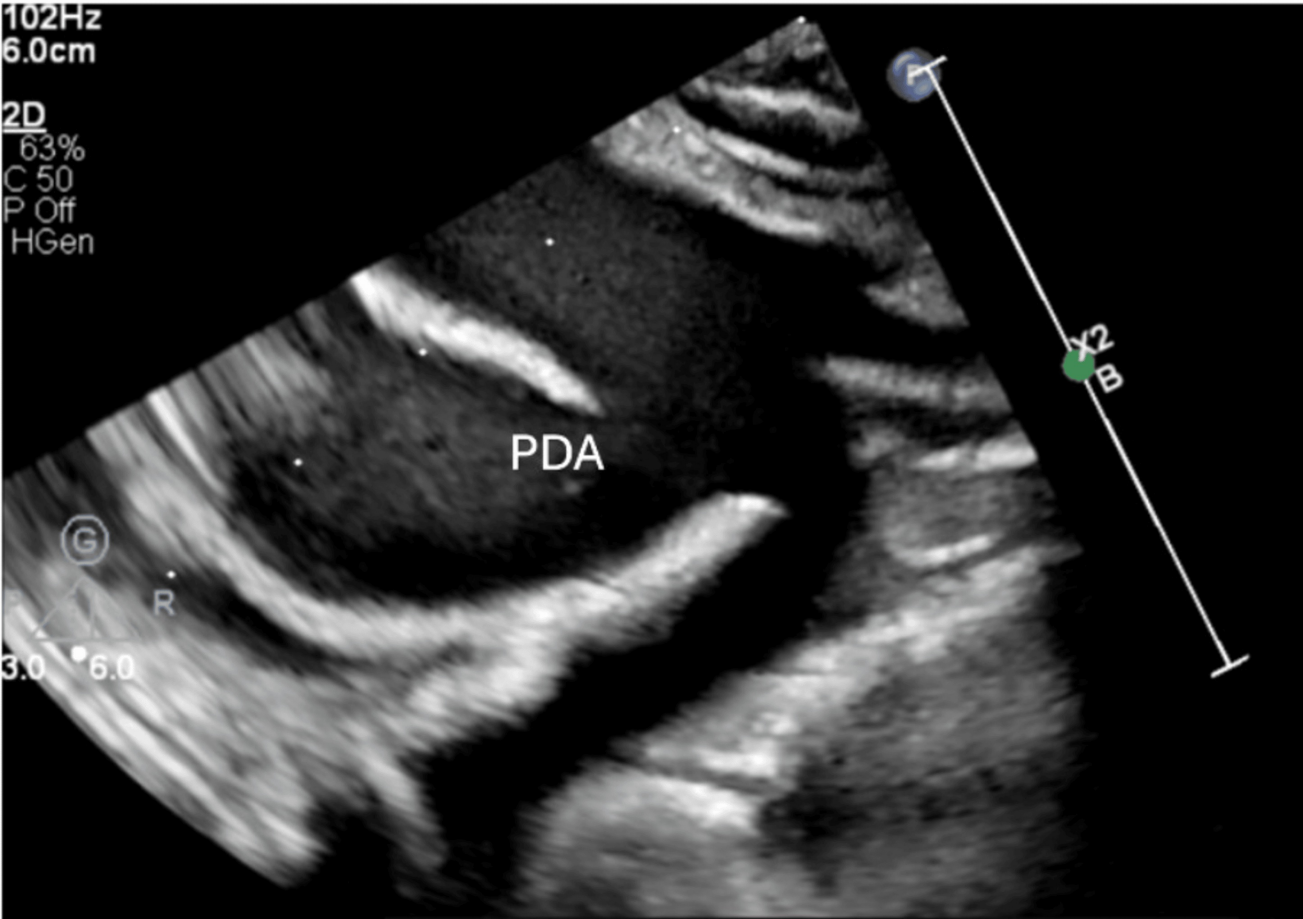
Window-type patent ductus arteriosus (PDA) with a normal left-sided aortic arch branching pattern.

**Figure 9:**
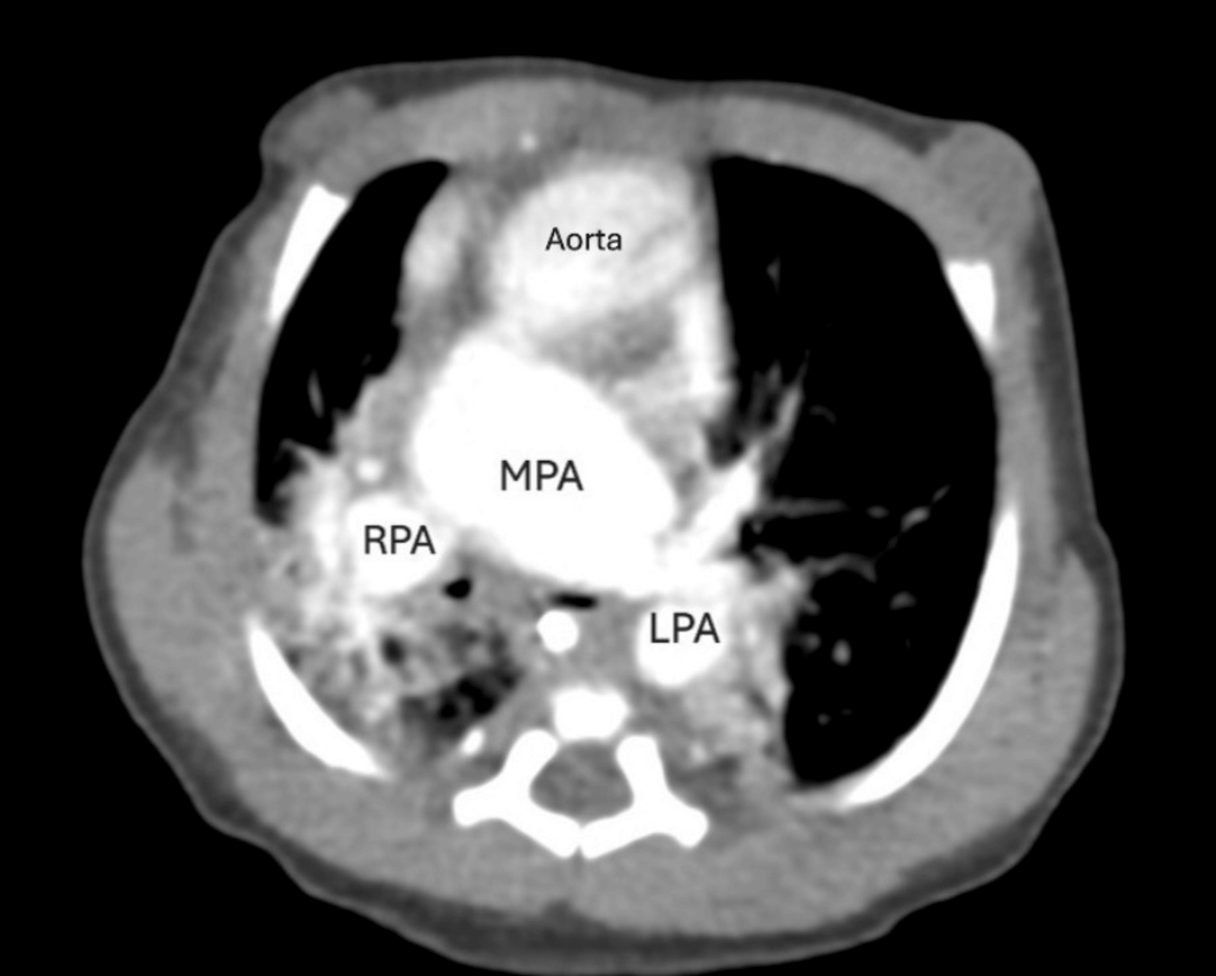
Axial contrast-enhanced CT angiography demonstrates a dilated main pulmonary artery (MPA) with clear bifurcation into the right pulmonary artery (RPA) and left pulmonary artery (LPA). Aorta is anterior to the MPA.

**Figure 10:**
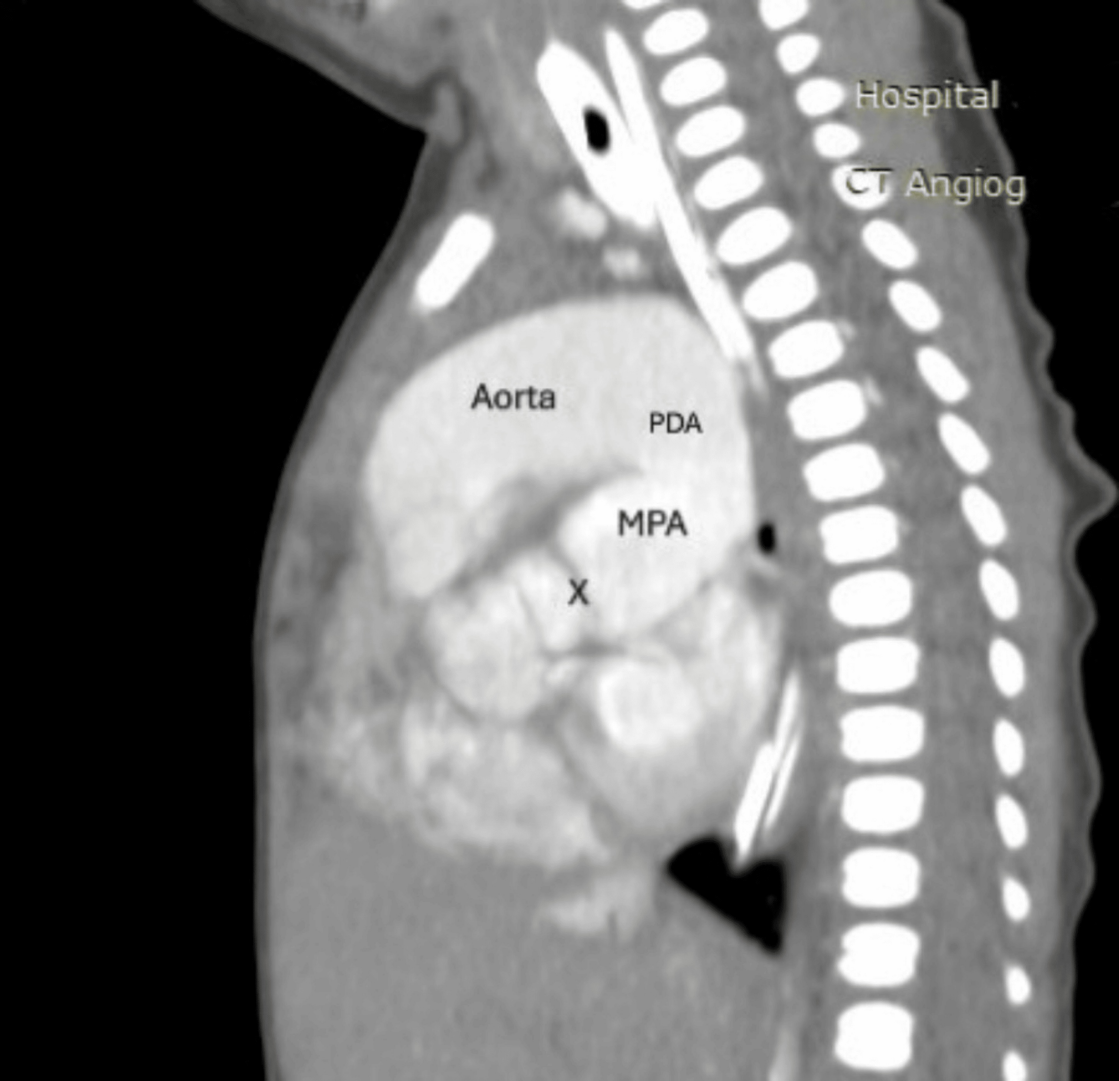
Sagittal contrast-enhanced CT angiography shows the dilated main pulmonary artery (MPA) positioned posterior to the transposed aorta, with a prominent patent ductus arteriosus (PDA) connecting to the descending aorta. The pulmonary valve is labeled as X.

Detailed echocardiography description

Echocardiography demonstrated classic features of TGA, with the aorta arising from the right ventricle and the pulmonary artery originating from the left ventricle. A large subaortic VSD permitted bidirectional flow. The pulmonary valve was severely dysplastic, rudimentary behaving like absent pulmonary valve, resulting in free pulmonary regurgitation and subsequent massive dilation of the main pulmonary artery. The branch pulmonary arteries were asymmetrical, with the left pulmonary artery measuring 3 mm (Z −2.15) and the right pulmonary artery 5 mm (Z=+0.94). A large window-type PDA measuring 8 mm exhibited bidirectional flow. The aortic valve was trileaflet but thickened, producing moderate stenosis with a mean gradient of 23 mmHg and trivial regurgitation. The ascending aorta measured 11 mm (Z=-1.2). There was mild tricuspid regurgitation, while the mitral valve appeared structurally normal. Ventricular function was preserved with a mildly dilated left ventricle.

Final Diagnosis

The final diagnosis is TGA, large VSD, dysplastic/absent pulmonary valve with free pulmonary regurgitation, moderate aortic valve stenosis, and a window-type PDA.

Management and outcome

Given the extraordinarily complex cardiac anatomy, the guarded surgical prognosis, and significant limitations in social support, including the challenges faced by very young parents with poor socioeconomic status, the case was comprehensively reviewed in a multidisciplinary meeting involving pediatric cardiology, cardiothoracic surgery, and neonatology teams. The family was counseled extensively regarding the overall poor prognosis, the anticipated need for multiple staged procedures, and the high risks associated with each treatment option. After a thorough understanding of the risks and anticipated outcomes, the parents ultimately elected for conservative management and palliative care. Consequently, the intravenous prostaglandin infusion was withdrawn, and the infant expired on day 10 of life.

## Discussion

Absent pulmonary valve syndrome (APVS) includes rudimentary, dysplastic, or absent pulmonary valve leaflets, dilated main pulmonary artery with or without dilatation of its branches, to-and-fro flow at the site of absent pulmonary valve, systolic pressure across the narrowed pulmonary valve [2,3]. The coexistence of TGA with APV is extraordinarily rare, with only isolated case reports documented [4]. APVS is typically associated with tetralogy of Fallot, where it accounts for approximately 3%-6% of cases [5,6]. When occurring with TGA, it represents a more severe disruption of conotruncal septation. Published literature describes similar anatomical constellations, though with considerable variability in clinical severity and surgical outcomes. Some infants, for instance, have presented with profound airway compromise due to the aneurysmal pulmonary arteries compressing the tracheobronchial tree, which often results in bronchomalacia and necessitates prolonged respiratory support.

As reported by Sakai et al., one infant with a similar diagnosis of TGA with APV underwent a palliative, staged approach including an anatomical left ventricle (LV) to pulmonary artery (PA) shunt, pulmonary artery plication, and tracheostomy while awaiting a subsequent Glenn shunt [2]. Conversely, Oppido et al. presented a similar case of TGA and APV which successfully underwent complete repair [7]. This patient also experienced severe airway compression secondary to the massively dilated pulmonary artery, but this complication resolved post-operatively. The current case, however, includes the concomitant presence of moderate aortic stenosis. This finding represents an exceptionally rare constellation for which no direct surgical references are currently available in the literature.

In the present case, the combination of severe pulmonary regurgitation, massive pulmonary artery dilation, and prominent ductal patency produced a hemodynamically unstable circulation dominated by pulmonary overcirculation. The large pulmonary root and branch dilatation raised the potential for airway compromise, consistent with prior reports, although its clinical expression may vary depending on airway caliber and lung compliance. Crucially, the coexistence of moderate aortic stenosis added an additional layer of complexity, contributing to systemic outflow obstruction and consequently impairing effective systemic perfusion. This unique and highly complex constellation of lesions significantly complicates the surgical decision-making process regarding the feasibility and sequence of repair, specifically, whether to address the pulmonary or aortic valve first, or if a complete repair is feasible in a single setting. Echocardiography plays a central role in early recognition, allowing differentiation from more common APV-TOF and ensuring appropriate counseling. In this case, the detailed imaging findings guided a multidisciplinary decision to pursue conservative care, recognizing the limited feasibility and expected poor outcome of surgical repair.

## Conclusions

This case highlights an exceptionally rare combination of TGA, VSD, absent pulmonary valve, and aortic valve stenosis, representing an extreme spectrum of conotruncal malformation. The early use of echocardiography was crucial for delineating the complex anatomy and informing clinical decision-making. The case emphasizes the importance of integrating hemodynamic severity, anticipated surgical complexity, and family considerations when determining the most appropriate management pathway. Ultimately, a multidisciplinary and compassionate approach remains central when faced with congenital cardiac anomalies associated with poor prognostic outcomes and limited interventional options.
